# Research advances in aptamers in the diagnosis and treatment of breast cancer (Review)

**DOI:** 10.3892/ijo.2026.5885

**Published:** 2026-04-21

**Authors:** Liqiang Chu, Yunfeng Xiao, Xinhua Li, Jiawei Cui, Luyao Ma, Bin Shao, Hongwei Cui

**Affiliations:** 1 School of Graduate, Inner Mongolia Medical University, Hohhot, Inner Mongolia Autonomous Region 010059, P.R. China; 2 Department of Scientific Research, Breast Cancer Center, Affiliated Cancer Hospital of Inner Mongolia Medical University/Peking University Cancer Hospital (Inner Mongolia Campus), Hohhot, Inner Mongolia Autonomous Region 010050, P.R. China; 3 School of Pharmacy, Inner Mongolia Medical University, Hohhot, Inner Mongolia Autonomous Region 010059, P.R. China; 4 Key Laboratory of Carcinogenesis and Translational Research (Ministry of Education), Department of Breast Oncology, Peking University Cancer Hospital and Institute, Beijing 100142, P.R. China

**Keywords:** aptamer, breast cancer, diagnosis, therapy, precision medicine

## Abstract

Cancer is a malignant disease that threatens human life and health. Among cancer types, breast cancer is one of the most common malignant tumors in women worldwide and ranks among the top cancer types in terms of incidence rate of malignant tumors in Chinese women. Aptamers are a class of DNA or RNA sequences that can bind to specific targets through their three-dimensional structures, and are screened and obtained via systematic evolution of ligands by exponential enrichment technology. Aptamers have advantages of high affinity and specificity, strong stability, low immunogenicity, a wide range of targets and ease of preparation and purification. As effective molecular targeting tools and drug delivery carriers, aptamers and their conjugates show therapeutic potential in the early diagnosis and treatment of breast cancer. The present article reviewed the properties and screening techniques of aptamers, as well as their applications and research progress in the diagnosis and treatment of breast cancer, with the aim of providing new strategies and directions for the realization of precision medicine for breast cancer via aptamers.

## 1. Introduction

A notable association exists between population aging and the disease burden of breast cancer. The incidence of breast cancer has steadily increased worldwide, thereby becoming a critical public health concern that poses a substantial threat to women's health (1). According to the 2022 edition of the Global Cancer Statistics Report, an estimated 2.297 million new breast cancer cases occurred worldwide in 2022, accounting for 11.6% of all malignant tumors in female patients. The global crude incidence rate of breast cancer is 58.7 cases per 100,000 individuals (2,3). In the same year, in China, the number of patients with breast cancer reached 357,000, accounting for 11.5% of the total number of patients with cancer in the country and 23.8% of all cancer cases among Chinese women. The incidence rate of breast cancer is second only to that of lung cancer, with 75,000 mortalities attributed to it. Breast cancer has become the second most prevalent malignant tumor globally, and both its incidence and mortality rates have been increasing at a rapid pace worldwide (4,5).

The development and progression of breast cancer, a type of hormone receptor-dependent tumor, are intricately intertwined with the abnormal expression of estrogen receptor (ER), progesterone receptor (PR) and human epidermal growth factor receptor 2 (HER2). The statuses of these biomarkers not only dictate the prognosis of the disease but also serve as a guide for formulating clinical treatment strategies (6,7). However, traditional detection methods, including radioimmunoassay, ELISA and immunohistochemistry, face difficulties in meeting the stringent requirements of precision medicine for biomarker detection. This is due primarily to issues such as poor antibody stability, complex operational procedures and challenges in cost management (8). Triple-negative breast cancer (TNBC), a subtype of breast cancer lacking ER, PR and HER2 expression, has limited treatment options, relying on chemotherapy, and has high recurrence and metastasis risks. Previous studies have shown dysregulation of immunogenic cell death (ICD)-related genes (such as CD8A and IFNG) in TNBC, which correlates with prognosis. TNBC is classified into immune 'cold' and 'hot' subtypes based on ICD genes, and an ICD score is developed to predict prognosis and immunotherapy response (9,10).

Aptamers are single-stranded oligonucleotides derived from systematic evolution of ligands by exponential enrichment (SELEX) technology (11). By exploiting their programmable three-dimensional spatial structures, such as stem loops and G-quadruplexes, aptamers demonstrate a high degree of specific binding ability to target molecules. Due to their antibody-like targeting and binding properties, aptamers are frequently dubbed 'chemical antibodies'. Compared with traditional antibodies, these 'chemical antibodies' possess distinct advantages, such as small molecular weights ranging from 10 to 30 kDa, excellent tissue penetrability, low immunogenicity and controllable chemical synthesis (12,13). These characteristics endow them with unique application value within the field of biomedicine.

Research on nucleic acid aptamers was initiated in the 1990s (13,14). Since then, they have been applied across a diverse spectrum of fields, predominantly but not confined to biosensing, molecular imaging, drug delivery, disease treatment and diagnosis. Particularly in the diagnosis and treatment of breast cancer, aptamers not only increase the efficacy of early diagnosis by increasing the sensitivity and specificity of liquid biopsy, but also serve a pivotal role as targeted drug delivery platforms in precision treatment strategies, including immunotherapy and gene therapy (12). With the continuous iterative advancement of SELEX technology and the innovative development of aptamer modification techniques, this class of molecular tools is gradually evolving from basic research to clinical translation. This transition offers a novel solution for precise diagnosis and personalized treatment of breast cancer.

## 2. Aptamers: Synergistic evolution of directed creation and functional properties

**Figure 1:**
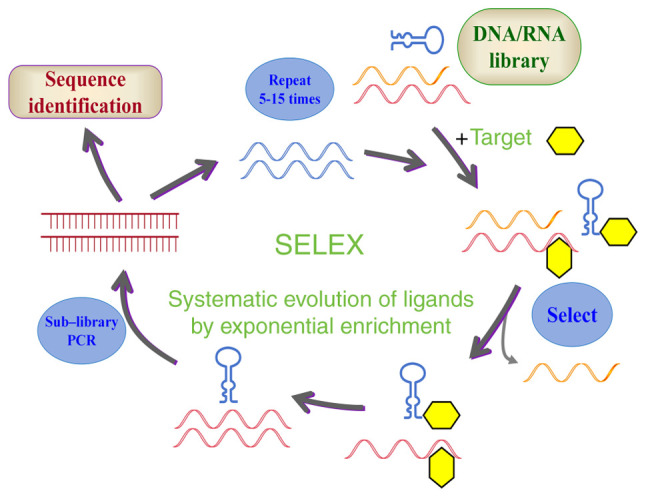
Schematic diagram of the SELEX process. Initially, a single-stranded DNA or RNA library containing numerous random sequences is synthesized. This library provides a vast array of structures capable of binding to various target molecules. Different techniques are subsequently utilized to monitor the binding of nucleic acid sequences to the target, enabling the isolation of specific aptamers. Next, the target-binding sequences are amplified via methods such as PCR, forming a secondary library for the next screening round. Finally, the screened sequences are sequenced, and through bioinformatics analysis, potential high-affinity aptamers are identified. SELEX, systematic evolution of ligands by exponential enrichment.

The core of aptamer development relies on SELEX technology, an *in vitro*-directed screening platform simulating Darwinian evolutionary principles (Fig. 1) (15-18). Previous foundational studies in 1990 first validated its feasibility: By constructing oligonucleotide libraries containing 1×10^13^ to 10^−15^ random sequences and subjecting them to iterative cycles of 'binding-elution-amplification,' the authors successfully screened DNA aptamers targeting T4 DNA polymerase and RNA aptamer-binding dyes (12,13).

**Figure 2:**
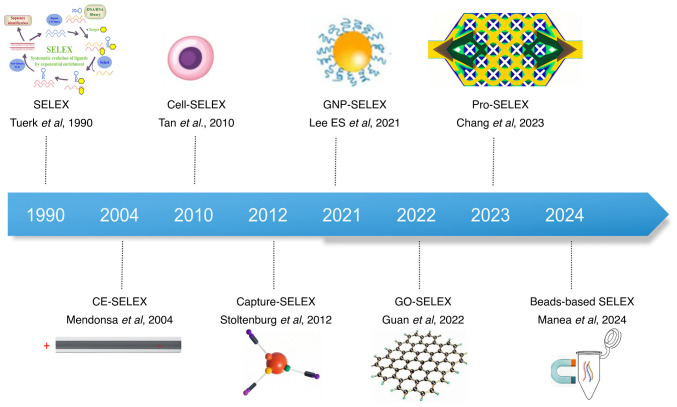
Timeline diagram of SELEX development. In 1990, Tuerk and Gold first introduced SELEX and successfully screened out aptamers (13,14). Over the subsequent 30-year period, SELEX has undergone continuous evolution. Various derivative technologies have emerged, including CE-SELEX (which utilizes capillary electrophoresis for screening), Cell-SELEX (targeting cells), capture-SELEX (for capturing biological macromolecules), GNP-SELEX (a gold nanoparticle-based screening method), GO-SELEX (based on graphene oxide), Pro-SELEX (employing parallel SELEX and high-content bioinformatics for aptamer selection) and magnetic bead-based SELEX (23-28). SELEX, systematic evolution of ligands by exponential enrichment; GO, graphene oxide; CE, capillary electrophoresis; GNP, gold nanoparticle.

The revolutionary value of this technology manifests in two key dimensions: First, the vast sequence libraries cover >99.9% of the theoretical conformational space (including hairpins, pseudoknots and G-quadruplexes), enabling target-specific binding via hydrogen bonds and base-stacking forces, with dissociation constants (Kd) reaching nM-pM levels (19-21). Second, the screening paradigm has continuously evolved: The Cell-SELEX developed by Sefah *et al* (22) in 2010 integrated positive/negative selection strategies, achieving, for the first time, the direct screening of live-cell-specific aptamers without prior knowledge of surface markers, thus revolutionizing traditional biomarker discovery pathways. Furthermore, technological advancements such as magnetic bead separation, Capillary Electrophoresis-SELEX (CE-SELEX) dynamic monitoring and nanopore single-molecule sequencing have reduced the number of screening cycles by 40-60% and enhanced the precision of CRISPR aptamer screening (Fig. 2) (23-28).

SELEX screening directly endows aptamers with a unique structure-function unity, which is further strengthened through engineering modifications. Their intrinsic properties include small size (10-30 kDa), enabling deep tissue penetration (5-fold greater than 150-kDa antibodies), reversible binding, supporting dynamic recognition, inherently low immunogenicity and a broad target spectrum (covering entities from ions to multilevel biological entities such as cells) (29). These properties significantly increase through engineering modifications: Phosphorothioate backbone modifications significantly increase nuclease resistance; 20-40-kDa PEGylation extends the serum half-life to ~6 h; pH-responsive DNA switches increase the affinity of the protein by 40% in the acidic tumor microenvironment (pH 6.5); and bispecific designs [HER2/epithelial cell adhesion molecule (EpCAM)] can increase target coverage to 85%..Chemical synthesis confers batch stability to >98% and enables controllable production costs, overcoming the biovariability bottleneck of antibody production shown (Table I) (30-33).

These technological characteristics synergistically drive transformative innovations in medical paradigms. In the diagnostic field, circulating tumor cell (CTC) capture aptamers screened by Cell-SELEX combined with nanopore sequencing achieve a detection sensitivity of 1 cell/ml. In the therapeutic field, the small size advantage enables delivery across the blood-brain barrier via lipid nanoparticles (LNPs), allowing targeted delivery of p53 aptamers to brain metastases, whereas the reversible binding property enables smart drug-controlled release systems [HER2 aptamer-doxorubicin (DOX) conjugates]. These applications ultimately converge into a personalized medicine closed loop: The 'Dynamic Aptamer Library' strategy, which is based on real-time monitoring of circulating tumor DNA (ctDNA), allows dynamic adjustment of PROTAC-aptamer conjugate combinations to combat tumor heterogeneity (34).

## 3. Applications of aptamers in the diagnosis of breast cancer

Aptamers, as cutting-edge molecular diagnostic agents, have considerable potential for precise breast cancer diagnosis due to their high target specificity, controllable synthesis and batch-to-batch stability. Critical for molecular classification, which relies on biomarkers such as ER, PR, HER2 and Ki-67 (35), aptamers enable diagnosis through two core strategies: i) Non-invasive liquid biopsy targets CTCs, extracellular vesicles (EVs) and cell-free nucleic acids in biological fluids, achieving high-sensitivity tumor marker detection while overcoming the invasiveness of tissue biopsies for dynamic early screening and treatment monitoring (36); and ii) targeted molecular imaging conjugate aptamers with fluorescent dyes, radionuclides or nanocontrast agents for selective tumor accumulation, significantly enhancing imaging efficacy. When integrated with magnetic resonance imaging (MRI) or PET, this approach surpasses traditional imaging limitations for <5-mm lesions (37). By optimizing sensitivity (detection limit, 0.5 nmol/l) and specificity (non-target binding, <5%), aptamer technology provides multidimensional solutions for precise staging, efficacy evaluation and recurrence monitoring. Its synergistic integration with traditional imaging methods (mammography and MRI) has propelled the diagnosis of breast cancer toward minimally invasive and intelligent paradigms.

### Applications of aptamers in liquid biopsy of breast cancer

Liquid biopsy, as a minimally invasive molecular diagnostic technology, provides dynamic assessment evidence for the early diagnosis, treatment stratification and efficacy monitoring of breast cancer by analyzing CTCs, cell-free DNA and EVs (38). The integration of nucleic acid aptamer technology in liquid biopsy is prominent in the detection of tumor-derived EVs. Leveraging microfluidic thermophoresis enrichment and nucleic acid aptamer recognition, highly sensitive detection of EV membrane proteins in small volumes of serum or plasma samples has become feasible (39).

The ctDNA detection indicators primarily consist of specific gene mutation sites (TP53, ESR1, PIK3CA and HER2 mutations corresponding to different breast cancer subtypes) and mutation abundance thresholds (quantified as MAF/VAF, with clinical decision thresholds of ≥1% for recurrence warning, ≤0.1% for favorable treatment response and a ≥2-fold increase for drug resistance), whose importance lies in non-invasively capturing tumor heterogeneity, guiding subtype-specific therapy selection, predicting treatment response and monitoring minimal residual disease. The lower limit of detection (LOD) is determined by technical validation (such as sequencing depth and background noise control via negative controls) and clinical background filtering (excluding CHIP mutations), while there is no strict upper limit, and clinical critical thresholds are established through retrospective/prospective cohort studies. Some cited indicators [TP53/ESR1 mutations in metastatic breast cancer (MBC), FDA-approved NGS panels for PIK3CA/ESR1 detection] have been clinically applied in prognosis stratification and treatment guidance; however, aptamer-mediated ctDNA detection and some mutations (KRAS) remain investigational, requiring further validation to avoid over-inferring the association between 'aptamer binding' and the clinical relevance of biomarkers (40-43).

The integrated technology combining microfluidic thermophoresis enrichment and aptamer recognition enables precise analysis of EV membrane proteins within nanoliter-scale serum samples. Tian *et al* (44) constructed a functional nucleic acid microfluidic sensor that exploits the thermophoresis effect induced by a temperature gradient to drive the directional migration of EVs. In conjunction with Cy5-labeled aptamer probes, this system boosts the EV enrichment efficiency to >5-fold that of conventional methods. The fluorescence signal amplification mechanism allows the quantitative detection of eight breast cancer-related EV protein markers. In a clinical study, 286 plasma samples from patients with MBC, non-MBC (NMBC) and age-matched healthy donors were subjected to thermophoretic aptasensor (TAS) measurements to obtain the profiles of eight EV markers. For instance, enzyme-linked immunosorbent assay (ELISA) was used to detect CD41 and CD63 on EVs isolated from the plasma of patients with MBC (n=5), patients with NMBC (n=5) and HD (n=5). Each group with n=5 represents a representative validation experiment within the larger cohort of 286 samples; it was demonstrated that EV protein markers measured by TAS can be used to distinguish MBC from NMBC (accuracy, 87.7%; 95% CI, 76.3-94.9%) (44,45).

Regarding the surface-specific antigens of CTCs, Gao *et al* (46) employed Cell-SELEX technology to screen a DNA aptamer, C24S, which specifically recognizes CD44+ cells. The fabricated magnetic nanoparticles enable the specific enrichment of CTCs under the influence of a magnetic field. This method capitalizes on the high-affinity binding (Kd=73.6 nM) between the aptamer and the CD44 molecule on CTC surfaces. When combined with magnetic separation technology, it elevates the capture efficiency of CTCs in blood samples to >85%, offering a non-invasive solution for detecting early tumor micrometastasis and monitoring recurrence.

Nucleic acid aptamers also serve to identify and detect specific proteins on the surface of or secreted by breast cancer cells, functioning as tumor markers. ER^+^ breast cancer accounts for >50% of all breast cancer cases and poses a severe threat to women's health. Cong *et al* (47) successfully screened a DNA aptamer, S1, capable of specifically recognizing ER^+^ breast cancer MCF-7 cells via Cell-SELEX technology. Further truncation and optimization yielded S1-4, which bind specifically to the membrane proteins of MCF-7 cells via a stem-loop structure. With a Kd of 73.6±7.5 nM, S1-4 exhibit robust binding capacity. The target of S1-4 was identified as a cell membrane protein, and this aptamer enables membrane protein recognition without cellular endocytosis. These properties render S1-4 promising molecular probes for the diagnosis and treatment of ER+ breast cancer, opening possibilities for the development of novel diagnostic approaches and treatment strategies. Additionally, the *in vivo* imaging application of S1-4 demonstrated its accumulation in MCF-7 tumor tissues, further validating its potential as a molecular probe.

The aforementioned study isolated a specific RNA aptamer (PRapt-3) targeting the DNA-binding domain (DBD) of progesterone receptor (PR), a key biomarker for breast cancer molecular typing, via an optimized CRUSOAK-SELEX strategy (eight SELEX cycles combined with a 'crush and soak' step to enrich target-bound RNA) (48). Notably, full-length PR consists of three functional domains (N-terminal domain, DBD and C-terminal ligand-binding domain) and exhibits inherent structural instability. This instability arises from the susceptibility of its multi-domain structure to conformational changes under physiological conditions (such as pH fluctuations, enzymatic hydrolysis or temperature variations in biological samples), which disrupts consistent binding with detection probes and compromises diagnostic accuracy. By contrast, the PR DBD is a highly conserved, compact functional domain with a stable three-dimensional structure that undergoes minimal conformational variation. By specifically targeting the PR DBD, PRapt-3 circumvents the binding interference caused by the structural instability of full-length PR, ensuring reliable and consistent recognition of the target. PRapt-3 exhibited high affinity (in terms of Kd and specificity for PR DBD, with no cross-reactivity to EGFR, IgE or human serum). Its secondary structure, featuring two hairpin loops and a multi-branched loop, enables specific binding to PR DBD. Clinically, PRapt-3 demonstrated significant diagnostic potential in breast cancer: It detected PR in enzyme-linked aptasorbent assay (LOD=200 nM), membrane-based assays (dot blot/western blotting, detecting 6.25 pmol PR DBD and full-length PR in MCF-7 cell lysates), aptacytostaining (nuclear staining of PR in MCF-7/HeLa cells) and aptahistostaining [specific nuclear staining of formalin-fixed paraffin-embedded (FFPE) breast cancer cell blocks and patient tissue sections]. Notably, PRapt-3 outperformed antibodies in FFPE samples due to its smaller size (~10 kDa) and superior tissue penetration, overcoming antibody failure caused by epitope cross-linking in fixed samples. These findings provided a novel molecular tool for PR detection in breast cancer, supporting precise subtype classification and diagnostic applications.

In TNBC, the absence of common hormone and HER2 receptors leads to the upregulation of certain biomarkers, suggesting new targets for TNBC diagnosis and treatment. The AptaB3/B5 aptamers screened by de Araújo *et al* (49) exhibit dose-dependent recognition of MDA-MB-231 cells, and cross-react with subtypes such as luminal A/B and HER2+, indicating their potential for detecting pan-breast cancer subtypes. In the future, it is anticipated that aptamer-based liquid biopsy will achieve minimally invasive breast cancer diagnosis.

Calreticulin (CRT), a protein closely associated with the onset of various cancer types, as has been reported to be significantly upregulated in oral, breast, gastric, colorectal, bladder, pancreatic, prostate and vaginal cancer. Among them, the role of CRT in breast cancer has garnered increasing attention (50-54). It is regarded as an effective novel biomarker for breast cancer invasion and metastasis, as well as an indicator of breast cancer stage and prognosis. Sun *et al* (55) utilized CE-SELEX technology to screen nucleic acid aptamers that specifically bind to CRT from a random single-stranded DNA library. After four rounds of screening, nucleic acid sequences with high affinity for CRT were successfully enriched. Through high-throughput sequencing and secondary structure analysis, Apt 23 was identified as the candidate aptamer with the lowest equilibrium Kd. With a Kd value of 670±56 nM, Apt 23 has a high affinity for CRT. Further experiments revealed that Apt 23, when fluorescently labeled with FAM, can detect CRT in serum via a laser-induced fluorescence detector. The detection limit of CRT in serum is as low as 0.5 nmol/l, and the detection range spans from 1 to 1,000 nmol/l. The specific binding of the fluorescently labeled probe to the surface of 4T1 cells validated the clinical significance of CRT as a marker for breast cancer invasion and metastasis.

EGFR, a transmembrane protein, is a key biomarker of breast cancer pathogenesis. Parihar *et al* (56) designed an aptamer sensor based on a cerium oxide (CeO_2_) and graphene oxide (GO) nanocomposite for non-invasive EGFR detection via electrochemical impedance spectroscopy (EIS). The CeO_2_-GO nanocomposite was synthesized via the sol-gel method and functionalized with EGFR-specific aptamers through covalent coupling. The quantitative analysis was carried out using a CeO_2_-GO nanocomposite-modified glassy carbon electrode (GCE), with significant changes in current observed upon binding of the target analyte. EIS analysis of the fabricated aptasensor (GCE/CeO_2_-GO/EGFR-Apt/BSA) revealed a broad linear detection range, from 10 fg/ml to 100 ng/ml. These findings underscore the high sensitivity and specificity of the aptamer sensor, as well as its potential for real-time, noninvasive EGFR monitoring in clinical samples (such as serum, sweat and saliva), thereby providing technical support for point-of-care real-time testing.

The minimally invasive characteristic of liquid biopsy (facilitating easy sample collection and repeatable sampling) synergizes with the precision of aptamer technology, increasing the sensitivity of tumor marker detection by 1-2-fold, and achieving a specificity >90% (57). The functional modification of aptamers (such as fluorescence labeling and nanoconjugation) continuously broadens the detection spectrum, driving the development of breast cancer diagnosis toward multiparameter and dynamic modalities and offering essential technical support for precision medicine. Aptamers and conventional methods show differences in the quantitative analysis of key biomarkers (Table II).

### Applications of nucleic acid aptamers in the imaging diagnosis of breast cancer

Mammography, which serves as the cornerstone for breast cancer screening, has substantially enhanced diagnostic efficacy with the integration of MRI and molecular imaging technologies (58,59). Through chemical modification strategies, such as biotin labeling, fluorescent dye conjugation or radionuclide incorporation, when combined with imaging modalities such as ultrasound and MRI, aptamers are emerging as pivotal molecular probes for the precise localization of tumor lesions (60,61).

The integration of aptamers with molecular imaging technologies has significantly improved the diagnostic accuracy for early-stage small tumor lesions. In the realm of medical imaging, the applications of aptamers are continuously expanding. By integrating multiple imaging techniques, the precision and sensitivity of tumor detection have been enhanced. For instance, the AS1411. A nanobubble (NB)-targeted imaging system developed by Fang *et al* (62) achieved dual-targeted ultrasound imaging of tumors and neovascularization through the covalent conjugation of the aptamer AS1411 with lipid NBs. This aptamer specifically recognizes nucleolin (NCL), which is highly expressed on the surfaces of both tumor and vascular endothelial cells. As a result, it prolongs the residence time of NBs at the lesion site to >3-fold compared with that of non-targeted probes. The experimental data revealed that AS1411. NBs maintain 60% of the signal intensity within 10 min after reaching the peak ultrasound intensity, whereas the signal of ordinary NBs decays by 80%. This targeted aggregation imaging property increases the detection rate of early tumors (with a diameter <5 mm) to 92%, significantly outperforming the 75% detection rate of traditional ultrasound.

Khajuria *et al* (63) developed an aptamer-modified multiwalled carbon nanotube (MWCNT-BA-APT) complex, which was engineered into an MRI-targeted contrast agent and a photothermal therapy platform via butyric acid functionalization and mucin-1 (MUC-1) aptamer conjugation. Material characterization demonstrated that the modified MWCNTs retained a complete carbon nanostructure, as verified by the carboxyl grafting detected through Fourier transform infrared spectroscopy and the observed hollow tube morphology via high-resolution transmission electron microscopy. Furthermore, the aptamers on its surface could specifically bind to the MUC-1 protein present on the surface of TNBC cells (MDA-MB-231). MRI scans indicated that, within the concentration range of 20-100 *μ*g/ml, the T2-weighted signal intensity of MWCNTs-BA-APT increased linearly, and its photothermal conversion efficiency reached 19.91 W/g, enabling precise imaging guidance at the tumor site (Table III).

Aptamer-mediated molecular imaging technology has overcome the detection limitations of traditional imaging for small lesions. Its targeting capability increases the tumor-normal tissue contrast by 2-3-fold, offering a reliable diagnostic foundation for early lesions with a diameter <1 cm. Ultrasound dual-targeted imaging enabled by AS1411. NBs and the MRI/photothermal synergistic platform of MWCNTs-BA-APT exemplify the potential of aptamers to evolve from single diagnostic applications to integrated diagnosis and treatment approaches. These advancements not only optimize the precise staging of breast cancer but also provide innovative solutions for intraoperative lesion localization and treatment efficacy assessment, propelling the development of imaging diagnosis toward molecular precision.

## 4. Applications of nucleic acid aptamers in the treatment of breast cancer

**Figure 3:**
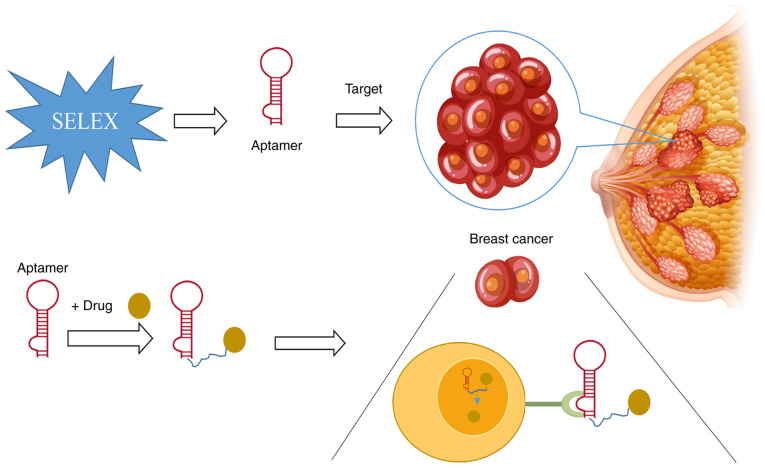
Schematic diagram of the mechanism of action of aptamers in the diagnosis and treatment of breast cancer. Aptamers, single-stranded DNA or RNA molecules obtained through SELEX screening, exhibit high specificity and can selectively recognize breast cancer tumor cells. When integrated with techniques such as liquid biopsy, these methods enable rapid tumor diagnosis. Furthermore, aptamers can function as drug carriers. By binding to therapeutic agents, they leverage their targeting properties to transport drugs to tumor sites, thereby enabling precise treatment. SELEX, systematic evolution of ligands by exponential enrichment.

Conventional treatment modalities, predominantly including chemotherapy, radiotherapy and surgery, suffer from insufficient targeting. This limitation not only fosters the rapid development of tumor cell resistance but also elicits severe systemic toxicity, resulting in irreversible damage to normal tissues (64). Additionally, the suboptimal efficiency of drug delivery exacerbates the risks of treatment failure and recurrence, underscoring the imperative for highly specific antitumor strategies. Nucleic acid aptamers, characterized by their programmable targeting and recognition capabilities, low immunogenicity and versatile chemical modification potential, are emerging as pivotal molecular tools for precise tumor treatment. These single-stranded oligonucleotides, which are isolated via SELEX technology, can selectively bind to biomarkers that are overexpressed on tumor cell surfaces, such as NCL and HER2 receptor. By doing so, they facilitate the precise delivery of antitumor drugs, significantly increasing drug accumulation in tumor tissues while minimizing the toxic effects on normal cells. Their unique molecular attributes, including nanoscale dimensions (10-30 kDa), tissue penetrability and chemical conjugability, offer an innovative approach to address the drug resistance and toxicity issues associated with conventional therapies. As depicted in Fig. 3, the applications of nucleic acid aptamers in tumor treatment span multiple facets, including targeted drug delivery, gene therapy and immunomodulation. The close alignment between their technical advantages and clinical requirements heralds substantial translational potential in the treatment of malignant tumors such as breast cancer.

### Aptamers as drug carriers

The aptamer-based targeted drug delivery (TDD) system, capitalizing on its precise molecular recognition capabilities, has emerged as an innovative strategy to overcome the limitations of traditional chemotherapy. Aptamers isolated via SELEX technology can specifically bind to tumor cell surface markers, facilitating the accumulation of drug carriers at lesion sites. This process significantly enhances the delivery efficiency and therapeutic index of antitumor drugs (65,66).

Tan *et al* (67) designed an aptamer-drug conjugate (ATC) that employs the AS1411 aptamer to target NCL, which is highly expressed in TNBC. Through receptor-mediated endocytosis, this conjugate enables the precise delivery of triptolide (TP). ATC specifically binds to NCL overexpressed on TNBC cell surfaces via AS1411 and enters cells through receptor-mediated endocytosis. In the acidic environment of the lysosome, the complex releases TP, inducing a change in lysosomal membrane permeability and triggering caspase-independent apoptosis. *In vivo* distribution experiments revealed that the fluorescence signal of ATCs in MDA-MB-231 tumor tissues persisted for 8 h, which is 4-fold longer when compared with the free aptamer. The tumor volume inhibition rate reached 62.3% (P<0.001), and no significant changes in mouse body weight were observed, confirming its low systemic toxicity.

Tang *et al* (68) constructed an HA1 aptamer-modified grapefruit-derived nanocarrier (GNV-Dox-HA1) that combines active targeting (HA1 recognizing HER2) and passive targeting [enhanced permeability and retention (EPR) effect] to achieve efficient DOX delivery (63). The HA1 aptamer, through its high-affinity binding to HER2 receptor, enables GNV-Dox-HA1 to specifically recognize and bind to HER-2-positive breast cancer cells such as MDA-MB-453 cells. The physicochemical properties of GNVs, including a particle size of ~200 nm and stable surface charge, endow them with excellent *in vivo* dispersibility and biocompatibility. Simultaneously, it achieves passive targeted delivery via the EPR effect. The drug loading efficiency of this system exceeded 90%, and it exhibited a controllable release profile at pH 7.4. The drug concentration in tumor tissues was 3-fold greater than that of free DOX. Experiments on tumor-bearing mice demonstrated that GNV-Dox-HA1 treatment results in a 78% tumor growth inhibition rate, extends the survival period by 40%, and reduces drug accumulation in the heart and liver by 60%, thereby significantly expanding the therapeutic window.

Rahmani *et al* (69) developed a chitosan (CS)-based nanocomposite for effective drug delivery to EGFR-positive breast cancer cells. This nanocomposite can inhibit breast cancer cell proliferation and induce apoptosis, with a synergistic effect observed between the aptamer and the drug NO. To create the nanocomposite, CS reacts with chloroacetic acid to introduce carboxyl (-COOH) groups, forming carboxylated CS (C-CS), which enhances its biocompatibility and surface functionalization. The DNA aptamer (Ap) was then covalently linked to C-CSs via EDC/NHS chemical coupling technology to form the Ap-C-CS nanocomposite. The aptamer Ap achieves targeted delivery to EGFR-positive breast cancer cells through its high-affinity binding to EGFR. The chemotherapeutic drug NO was loaded into the Ap-C-CS nanocomposite to form Ap-C-CS-NO. Techniques such as dynamic light scattering and transmission electron microscopy were employed to verify the particle size (~50 nm), morphology and surface charge (+27.7 mV) of the nanocomposite, ensuring its stability and targeting ability under physiological conditions. Aptamer Ap inhibits tumor cell proliferation by blocking the EGFR signaling pathway, whereas NO induces apoptosis by interfering with tubulin polymerization and activating the mitotic checkpoint. Additionally, NO inhibits tumor angiogenesis by downregulating the expression of hypoxia-inducible factor 1α and vascular endothelial growth factor, thereby enhancing the antitumor effect.

Taghipour *et al* (70) designed the SRL2/TA1 dual-aptamer conjugated micelle (MST@PBAS), which enables tumor microenvironment-responsive release of docetaxel (DTX) by targeting the LRP-1/CD44 dual pathway. A poly(β-amino ester) (PBAS) polymer containing disulfide bonds was synthesized through the reaction of 1,4-butanediol diacrylate, bis(2-acryloyloxyethyl) disulfide and 3-amino-1-propanol in dichloromethane. The camouflaged micelles were subsequently prepared via treatment with ethylenediamine and modification with MAL-PEG-NHS. The DTX-loaded MST@ PBAS micelles exhibited a uniform spherical structure with a particle size of ~100 nm and a surface charge ranging from −14.5 to −6.20 mV, meeting the criteria for an ideal nanodrug delivery system (namely, particle size <100 nm and neutral or negative surface charge). The SRL2 peptide promotes transcellular transport of micelles by targeting LRP-1, whereas the TA1 aptamer enhances cellular uptake by targeting CD44. This dual-targeting strategy significantly improves micelle accumulation in tumor tissues and intracellular drug release. In the tumor microenvironment, under the action of the MMP-9 enzyme, the micelle camouflage layer is removed, exposing the SRL2 and TA1 aptamers for precise targeting. In the presence of high glutathione and low pH, the micelle structure disintegrates, rapidly releasing DTX and overcoming multidrug resistance (MDR). The meticulously designed nanostructure in that study demonstrated superior anticancer efficacy by enhancing tumor targeting, enabling more accurate drug delivery, promoting drug release, reducing treatment dosage and minimizing anticancer drug side effects.

### Aptamer-mediated gene therapy

Small interfering RNA (siRNA)-mediated gene silencing therapy has emerged as a key avenue for the precise treatment of breast cancer, primarily by specifically inhibiting the expression of oncogenes. However, challenges such as the poor *in vivo* stability of siRNAs and the low efficiency of targeted delivery have spurred the development of aptamer-functionalized nanocarriers. Owing to their exceptional targeting capabilities, nucleic acid aptamers have emerged as indispensable tools for enhancing the specificity and efficiency of siRNA delivery. They have also shown unique advantages in gene silencing processes associated with tumor immune escape and suicide gene therapy (71). Furthermore, combination therapies involving siRNAs targeting multiple pathways, when used in conjunction with traditional chemotherapeutic drugs, have exhibited synergistic effects in numerous preclinical studies. This has opened novel treatment options guided by personalized and precision medicine principles for patients with breast cancer.

Camorani *et al* (72) fabricated sTN145 aptamer-modified nanoparticles [poly(lactic-co-glycolic acid) (PLGA)-b-PEG/sTN145] through a double-emulsion method, encapsulating programmed death-ligand 1 (PD-L1) siRNA within the hydrophilic core to enable the precise recognition of TNBC cells. In the aforementioned study, the PLGA-b-PEG copolymer served as the base material, and the nanoparticles were prepared via the water-in-oil-in-water (w1/o/w2) double-emulsion technique. This preparation approach effectively encapsulates siRNA within the hydrophilic core of the nanoparticles, safeguarding it from degradation. Aptamer-mediated receptor endocytosis significantly increased the uptake efficiency of these nanoparticles in MDA-MB-231 cells, increasing it by 3-fold compared with that of nontargeted carriers. Treatment markedly reduced the PD-L1 protein level within 90 min, resulting in an inhibition rate of 65% (P<0.01), and continuously suppressed the expression of the Snail transcription factor, which is associated with epithelial-mesenchymal transition, by 40%. This action blocks tumor invasion and metastasis pathways at the molecular level.

Taghizadeh-Tabarsi *et al* (73) developed the GOQD-PEI/S2.2 complex, leveraging the S2.2 aptamer (with a Kd of 0.1 nM, which has an affinity 1,000-fold greater than that of the traditional MA3 aptamer), to specifically bind to the MUC-1 glycoprotein on the surface of MCF-7 cells. This binding mediates the targeted delivery of the inducible caspase-9 (iC9) suicide gene (73). The key innovation of this complex lies in the targeted conjugation of the S2.2 nucleic acid aptamer. The aptamer specifically recognizes the MUC-1 glycoprotein, which is overexpressed in breast cancer cells, through its unique nucleic acid folding structure (Kd=0.1 nM), with its affinity enhanced by 3-fold compared with that of the traditional MA3 aptamer. In the organoid coculture model, the complex initiates endocytosis via a conformational change. Compared with the control, the dimerization of iC9 induced by AP1903 activated the caspase-9-dependent apoptosis pathway, resulting in a 72% cell cycle arrest rate in the G_2_/M phase and a 55% increase in the proportion of mitochondrial membrane potential collapse (P<0.001). These results demonstrate its superior targeted elimination ability compared with that of the two-dimensional culture model.

Gharehbaba *et al* (74) designed MUC-1 aptamer-modified Janus nanoparticles (Ap-As-JNPs) that integrate chemotherapy, photothermal therapy and gene therapy, achieving efficient reversal of MDR through a pH-responsive CS coating. In that study, dumbbell-shaped Ag/mesoporous silica nanoparticle (MSN) JNPs were synthesized via the sol-gel method. These nanoparticles had an average particle size of 285±5.12 nm, and were surface modified with amino and COOH functional groups, followed by further functionalization with a CS coating. The CS coating acts not only as a pH-responsive 'gating' molecule to prevent premature drug release but also as a positively charged platform for the binding of antisense molecules and aptamers. The 285-nm JNPs were loaded with DOX at a loading efficiency of 85.2% and P-gp antisense molecules. In the slightly acidic tumor microenvironment (pH 5.4), the cumulative drug release rate reached 77.6% within 72 h, which was significantly greater than the 11.4% release rate in a neutral environment. This pH-responsive release mechanism ensures efficient drug delivery to tumor tissues while minimizing toxicity to normal tissues. The photothermal conversion efficiency of JNPs is realized through the surface plasmon resonance effect. Upon irradiation with an 808-nm laser, the photothermal effect reduced the survival rate of MCF-7 cells to 30.05%, whereas this value was 39.11% in the group treated with only DOX. The photothermal effect not only enhances the cytotoxicity of DOX but also disrupts cell membrane integrity through local heating, thereby further improving drug uptake efficiency. The MUC-1 aptamer significantly increased the cellular uptake rate of JNPs by specifically binding to the MUC-1 receptor overexpressed on the surface of MCF-7 cells. Flow cytometry analysis revealed that the uptake rate of Ap-As-JNPs by MUC-1-positive MCF-7 cells reached 53.5%, whereas that by MUC-1-negative MDA-MB-231 cells was only 19.4%. Furthermore, the antisense P-gp molecule inhibits the expression of the P-gp pump by hybridizing with MDR1 mRNA, achieving an 80% inhibition rate of P-gp protein expression and restoring the sensitivity of drug-resistant cells to DOX to the level of wild-type cells.

Collectively, the aforementioned studies underscore the value of aptamers in gene therapy. By specifically recognizing tumor markers such as MUC-1 and PD-L1, aptamers can increase the delivery efficiency of siRNAs or suicide genes by 2-3-fold while reducing off-target effects. The ultrahigh affinity of the S2.2 aptamer (with a Kd as low as 0.1 nM) and the multimodal response mechanism of JNPs offer a reusable technical framework for overcoming tumor heterogeneity and drug resistance. With the integration of organoid models and precise delivery systems, aptamer-mediated gene therapy has evolved from single-gene silencing to multipathway coordinated regulation and has become a vital pillar in the personalized treatment of breast cancer.

### Aptamer-mediated immunotherapy

Immunotherapy has emerged as an important milestone in modern oncology, revolutionizing the treatment paradigms for various solid tumors. This therapeutic approach encompasses a diverse array of strategies, including immune checkpoint inhibitors, cancer vaccines, antibody-drug conjugates (ADCs), oncolytic viruses and adoptive cell therapies (75). Among these, immune checkpoint inhibitors have been the most extensively investigated immunotherapeutic agents for treating breast cancer (76,77). However, the limited efficacy of single-agent therapy, which is primarily attributed to tumor heterogeneity and immune escape mechanisms, has propelled the exploration of combination strategies that integrate targeted aptamers with immunotherapy, thereby establishing this area as a prominent research focus. Nucleic acid aptamers serve a pivotal role in augmenting the activity of immune cells and synergistically blocking immune checkpoints. They achieve this goal by targeting tumor-specific antigens or remodeling the immune microenvironment (78).

Camorani *et al* (79) developed the Gint4. T aptamer, which targets platelet-derived growth factor receptor β (PDGFRβ), which is highly expressed in TNBC. This aptamer forms a synergistic therapeutic system in conjunction with an anti-PD-L1 monoclonal antibody. PDGFRβ, a transmembrane receptor tyrosine kinase, is highly expressed in TNBC, particularly in invasive tumor cells exhibiting a mesenchymal/stem cell phenotype. It promotes tumor cell proliferation, migration and invasion by activating the downstream PI3K/Akt and ERK1/2 signaling pathways. The Gint4. T aptamer (with a Kd of 57.48 nM in human TNBC cells) inhibits tumor cell proliferation by blocking the PDGFRβ/PI3K-Akt signaling pathway, resulting in a single-aptamer inhibition rate of 25%. When combined with an anti-PD-L1 monoclonal antibody, it significantly enhances antitumor activity, with the combination treatment achieving a 50% inhibition rate compared with 37% for the monoclonal antibody alone. The anti-PD-L1 monoclonal antibody blocks the interaction between PD-L1 and PD-1/B7.1 receptors, thereby alleviating immunosuppressive signals within the tumor microenvironment and enhancing the T-cell-mediated antitumor immune response. Mechanistic studies indicated that the combination treatment increased interleukin IL-2 secretion by lymphocytes by 28%, increased interferon-γ release by 35%, and significantly increased T-cell-mediated cytotoxicity, as evidenced by a 40% increase in lactate dehydrogenase release. *In vivo* experiments confirmed that this strategy inhibits the growth of MDA-MB-231 tumors by 50% and reduces the number of lung metastasis nodules by 45%, underscoring the dual advantages of targeted pathway inhibition and immune activation (74). However, the efficacy of single immune checkpoint inhibitors in TNBC remains limited, mainly because of the presence of immunosuppressive factors in the tumor microenvironment, such as the infiltration of FOXP3^+^ regulatory T (Treg) cells and tumor cell immune escape mechanisms.

Chen *et al* (80) synthesized and labeled the PDGC21-T aptamer, linked it to a double C18 hydrocarbon chain, and incubated it with natural killer (NK)-92 cells to generate aptamer-engineered (ApEn)-NK cells. Treatment with ApEn-NK cells induced the apoptosis and death of TNBC cells but had a minimal effect on non-targeted breast cancer cells. Notably, treatment with the aptamer alone did not affect tumor cell apoptosis or death. Additionally, this treatment significantly inhibited the lung metastasis of TNBC cells. This study represents a novel approach to targeted immunotherapy for TNBC through the use of ApEn NK cells. An aptamer-anchor structure was formed by linking the Cy3-labeled PDGC21-T aptamer to a double C18 hydrocarbon chain. This structure inserts into the membrane of NK-92 cells via hydrophobic interactions, exposing the hydrophilic aptamer portion on the cell surface and thereby enabling targeted recognition of TNBC cells. The 1 *μ*M aptamer-anchor structure can efficiently modify NK cells within 30 min of incubation at 37°C, with an anchoring efficiency exceeding 90%, without compromising cell proliferation. ApEn-NK cells recognize an unknown target on the surface of TNBC cells through the aptamer. When the effector-to-target ratio was 1:3, the tumor cell apoptosis rate induced by ApEn-NK cells reached 45%, which was significantly higher than the 28% rate observed in unmodified NK cells, but had no significant effect on MCF-7 cells (with an apoptosis rate of <15%). In a lung metastasis model, the number of metastatic nodules in the ApEn-NK treatment group decreased by 60%, confirming that aptamer modification can enhance the tumor tropism and killing specificity of NK cells (80).

Macrophages are integral to the body's anticancer defense mechanisms, and participate in tumor cell phagocytosis, antigen presentation and adaptive T-cell activation. Yang *et al* (81) constructed engineered macrophages, designated CAM1, by anchoring a cholesterol-modified AS1411 aptamer on the surface of M1 macrophages, aiming to enhance tumor immunotherapy. This aptamer enables the selective targeting and efficient phagocytosis of tumor cells, promotes the secretion of anti-inflammatory cytokines and remodels the tumor microenvironment. It has potent antitumor effects both *in vitro* and *in vivo*, and exhibits favorable biosafety, suggesting that it is a novel strategy for tumor immunotherapy. The AS1411 aptamer specifically binds to nucleic acid-binding proteins on the surface of tumor cells, such as P190B, inducing tumor cell apoptosis and immunogenic death. Simultaneously, it promotes the infiltration and activation of immune cells, enabling CAM1 cells to achieve a breast cancer cell phagocytosis rate of 78% (at an effector-to-target ratio of 10:1), which is 2.3-fold greater than that of unmodified M1 cells. Furthermore, AS1411 enhances the antigen-presenting ability of macrophages, resulting in a 50% increase in CD8^+^ T-cell infiltration, a 35% decrease in FOXP3^+^ Treg cells and a significant increase in proinflammatory factor secretion, including a 60% increase in IL-1β and a 45% increase in TNF-α. *In vivo* experiments revealed that CAM1 treatment resulted in a 58% tumor volume inhibition rate with no apparent systemic toxicity, suggesting an innovative strategy for overcoming the tumor immunosuppressive microenvironment. Additionally, AS1411 can enhance the phagocytic function and antigen-presenting ability of macrophages by binding to specific receptors on the surface of M1 macrophages.

Collectively, the aforementioned studies elucidate the central role of aptamers in immunotherapy. By targeting tumor drivers, such as PDGFRβ, to relieve immunosuppressive pathways or by modifying the surface of immune cells to enhance targeting, as demonstrated by ApEn-NK and CAM1, aptamers can increase the response rate of immunotherapy by 20-30%. The dual-pathway regulation of Gint4. T, the directional modification of NK cells by PDGC21-T and microenvironment remodeling by AS1411 highlight the technical versatility of aptamers, which transition from single-targeting to immune network regulation. With the increasing prominence of CAR-NK cells and engineered macrophage therapies, aptamer-mediated precise targeting has become a key technology for optimizing the efficacy-safety balance of immunotherapy, driving the evolution of breast cancer immunotherapy from 'extensive activation' to 'precise regulation'.

### Comparative studies of aptamers and other carriers

In the field of tumor-targeted therapy, nucleic acid aptamers serve as a functional complement to various delivery systems, including traditional antibodies, viral vectors, liposomes and polymer nanoparticles. The distinct characteristics of these entities drive the optimized selection of precision medicine strategies. Aptamers and antibodies, two central tools for targeted therapy, exhibit complementary features in tumor treatment. Therapeutic antibodies, which leverage the antibody-dependent cellular cytotoxicity and complement-dependent cytotoxicity effects mediated by the Fc segment, demonstrate remarkable efficacy in indications such as HER2^+^ breast cancer, with their affinity reaching the pM level (Kd<1 pM) (82). However, their relatively large molecular weight of 150 kDa results in limited penetration efficiency within solid tumors. For example, the anti-PSMA antibody predominantly accumulates around tumor blood vessels, with an interstitial penetration depth of <50 *μ*m (83). By contrast, the serum half-life of aptamers (molecular weights ranging from 10 to 30 kDa) can be extended to 6-12 h through modifications such as 2'-fluorine modification or phosphorothioate backbone optimization, enabling them to rapidly diffuse into deeper regions of tumors. Taking the PSMA aptamer A9 as an example, its targeted delivery mediated by a three-dimensional conformation increases the tumor-to-normal tissue drug concentration ratio by 3-fold, significantly outperforming antibody carriers (84). In terms of drug-loading capacity, a unified drug-to-carrier molar ratio is adopted for accurate comparison: Aptamers directly conjugated with drugs have a drug-to-aptamer molar ratio of 1:1-4:1 (corresponding to a mass percentage of <10% for small-molecule drugs), which is lower than the drug-to-antibody ratio (DAR) of ADCs (3:1-8:1, with site-specific conjugation ADCs typically having a uniform DAR of 4:1). For aptamer-functionalized nanocarriers, the total drug-to-aptamer molar ratio can reach 10^3^:1-10^5^:1 (calculated by dividing the total encapsulated drug molecules by the number of aptamers on the carrier surface), which is 1-3-fold higher than that of ADCs, although this ultra-high loading is attributed to the nanocarrier's internal cavity, with aptamers only serving as targeting ligands. Despite the lower drug-loading capacity of directly conjugated aptamers compared with that of ADCs, their controllable chemical synthesis process and production cost (1/10th that of antibodies) make them ideal for combination therapy. The molecular weight of aptamers and antibodies differs 5-15 times. Using mass ratio as an evaluation index will distort results due to carrier mass differences, while the D/C ratio, based on drug molecules per carrier molecule, is independent of molecular weight, truly reflecting drug-loading efficiency (85-88). Adopting the D/C ratio as the core index eliminates interference from varying carrier molecular weights and drug-loading strategies, enabling direct and accurate comparison of drug-loading capacities of targeted delivery systems like aptamers and ADCs.

Adeno-associated virus (AAV) and lentiviral vectors rely on natural infection mechanisms, enabling a gene delivery efficiency of 70-80%. However, the T-cell responses triggered by AAV capsid proteins, which occur with an incidence of 15-20%, and the risk of lentiviral integration mutation, with a probability of ~0.1%, limit their clinical application (89). The chemical synthesis nature of aptamers circumvents the risk of biological contamination. Modifications such as 2'-O-methylation or PEGylation can increase their *in vivo* stability by 5-10-fold, making them more suitable for long-term administration. For example, an aptamer modified with locked nucleic acid has a half-life of >48 h in cynomolgus monkeys, which is notably improved in comparison with the 6-8 h of viral vectors (90). Additionally, the modular design of aptamers allows their combination with the CRISPR-Cas system to construct an integrated platform featuring 'targeted editing-responsive release'. By contrast, the functional modification of viral vectors is constrained by the structural diversity of capsid proteins (91).

Liposome carriers can prolong their circulation time to >12 h by adjusting the cholesterol ratio within the range of 20-50%. However, their passive targeting mechanism relies on the EPR effect, and their effectiveness in poorly vascularized tumors is limited (92). Aptamer-modified liposomes, through covalent conjugation with the HER2 aptamer, increase the delivery efficiency of DOX by 60%, with uptake by HER2^+^ cells reaching 250 fmol/10^6^ cells and reducing liver accumulation by 30% (93). In a TNBC model, this active targeting strategy increased the tumor growth inhibition rate from 45% (for non-targeted liposomes) to 72% and reduced cardiotoxicity by 50%. The pH-responsive modification of aptamers, such as the incorporation of acid-sensitive phosphodiester bonds, further enables the controllable release of drugs in the tumor microenvironment (pH 6.5), with the release rate being 4-fold higher than that in a neutral environment (pH 7.4) (94,95).

Polymer carriers such as PLGA can achieve a drug loading efficiency of >40% because of their adjustable particle size (ranging from 50 to 200 nm) and surface charge (from −20 to + 30 mV). However, their targeting ability depends on the modification of antibodies or peptides (96). The introduction of aptamers increases the specificity of these carriers by 2-3-fold. For example, MSNs modified with the MUC-1 aptamer exhibit an uptake rate of 53.5% by MCF-7 cells, which is 15% higher than that of the antibody-modified group (97). The combination of nanocarriers (such as gold nanoparticles and mesoporous silica) and aptamers has become a prominent research area. When combined with the photothermal effect, the aptamer-graphene quantum dot complex enables the integration of PD-L1 detection (with a detection limit of 0.5 nM) and tumor ablation (reducing cell viability to 30%) under irradiation with an 808-nm laser, demonstrating the characteristics of logic gating release (dual response to pH/temperature) (98). The size uniformity of this nanocomplex (with a coefficient of variation <10%) and the flexibility of the modification sites (allowing for controllable conjugation at the 5'/3' ends) make it a programmable platform for precision medicine (99).

The collaborative innovation between aptamers and delivery systems centers around three main directions: i) Enhancing the recognition of tumor heterogeneity through dual-targeted aptamers (such as the tandem of HER2/EpCAM), reducing the off-target rate to <5%; ii) developing aptamer-viral vector hybrid systems, which utilize aptamers to shield viral capsid antigens and reduce immunogenicity by >70%; and iii) constructing aptamer logic circuits that respond to multiple signals (pH/enzyme/temperature) in the tumor microenvironment to achieve precise spatiotemporal control of drug release. These technological breakthroughs are propelling the evolution of targeted therapy from 'single carriers' to 'intelligent complexes', paving the way for the individualized treatment of solid tumors, including breast cancer (Table IV).

## 5. Discussion and prospects

Nucleic acid aptamers, characterized by their high specificity, have emerged as an optimal choice for precision medicine, offering personalized treatment regimens for patients, and demonstrating substantial potential in cancer diagnosis and treatment research (100). Leveraging technologies such as liquid biopsy, nucleic acid aptamers contribute to the early detection of major diseases, including breast cancer. This early intervention considerably enhances treatment success rates and patient survival outcomes. Furthermore, as biomarkers, they enable real-time monitoring of treatment efficacy, providing a scientific basis for timely adjustment of therapeutic strategies. As programmable molecular tools, nucleic acid aptamers also help reduce medical costs and improve the economic viability of treatment. With technological advancements, the number of nucleic acid aptamer-based candidate drugs in clinical trials is steadily increasing, underscoring their immense potential for clinical translation. The aptamer-based TDD system has emerged as a highly promising approach for treating various diseases, including cancer. Recent progress in this field has facilitated the development of diverse aptamer-based TDD platforms. Due to their unique properties, nucleic acid aptamers represent an attractive alternative to other ligands in TDD, since they can specifically recognize cell surface targets. As chemically synthesized analogs of antibodies, nucleic acid aptamers possess several advantages over traditional antibodies in therapeutic development, which has spurred interest in developing them as substitutes for nucleic acid-based antibody and peptide ligand formulations.

Despite their promise as novel therapeutic and diagnostic tools with high affinity and specificity, nucleic acid aptamers face notable challenges during clinical translation, including difficulties in large-scale production and high-purity separation via solid-phase synthesis technology, issues related to the pharmacokinetics and biodistribution inherent to macromolecular drugs, safety and stability concerns stemming from potential immunogenicity, and risks of off-target and nonspecific binding during *in vivo* targeting (101). To address these issues, the development of high-throughput and cost-effective screening technologies, precise molecular engineering strategies, and safe and efficient drug carrier systems, such as ferritin nanocages, is imperative. Despite these challenges, the clinical application of nucleic acid aptamers continues to advance. For example, photosensitive molecular targeted drugs developed through precise molecular engineering strategies have shown potential in enhancing the efficacy of cancer photodynamic therapy (102).

Insufficient stability remains a central challenge. Unmodified aptamers exhibit a short half-life of only a few min in serum, which limits the maintenance of effective concentrations *in vivo* (103-105). Additionally, aptamer stability is influenced by environmental factors such as pH, temperature and ionic strength, which can induce structural changes and impair their functionality. Substituting the phosphodiester bonds of nucleic acid aptamers with phosphorothioate bonds can significantly increase their resistance to nuclease degradation. Although modification of the phosphorothioate backbone can extend the plasma stability by 3-fold (the half-life of S-XQ-2d reaches 6 h), complex modifications may compromise target binding activity (106,107). The issue of targeted escape due to tumor heterogeneity cannot be overlooked; ~30% of patients with HER2^+^ breast cancer exhibit an inadequate response to single-target aptamers due to antigen expression heterogeneity (108-110). Designing multivalent aptamers (such as the dual-targeting of HER2/EpCAM) can increase target coverage to >85% through synergistic binding. Multivalent aptamers increase the cell surface residence time via steric hindrance and can recognize antigen clusters on tumor cell surfaces, thereby reducing the probability of heterogeneity-induced escape. Inserting pH-sensitive DNA motifs into aptamer sequences enables the design of molecular switches with pH-selective binding properties. When combined with pH-responsive molecular switches, the targeting affinity can be enhanced by 40% in the slightly acidic tumor microenvironment (pH 6.5) (27,111).

Immunogenicity and off-target toxicity poses another critical challenge. Although aptamers generally exhibit lower immunogenicity than antibodies do, repeated administration may trigger the production of anti-aptamer antibodies (occurring in ~15% of cases), leading to a decline in therapeutic efficacy (112,113). Preclinical studies have reported that aptamers can activate the complement system in breast cancer models, causing local inflammatory reactions (114-116). Furthermore, aptamer-conjugated drugs (such as chemical agents or radionuclides) may damage normal tissues via off-target effects, particularly those that pose potential toxicity to organs with low-level target expression, such as the heart or liver (117). Preclinical evidence indicates that nonspecific accumulation of aptamer-conjugated drugs in the liver and kidneys can elicit toxic reactions. PEGylation with a molecular weight of 20-40 kDa is often required to reduce the clearance rate by the reticuloendothelial system (RES) by 60% while preserving target binding activity (118).

Delivery efficiency and uneven biodistribution also impedes treatment efficacy. The *in vivo* distribution, metabolism and excretion of aptamers are complex processes influenced by various factors, including molecular size, charge and interactions with biomolecules (119). Although aptamers can serve as targeted components of drug delivery systems, their delivery efficiency is hampered by multiple factors. The monodispersity of nucleic acid aptamers renders them susceptible to clearance by the reticuloendothelial system (RES) during *in vivo* delivery, and their cell membrane permeability is limited. The integration of nanocarriers has significantly mitigated these limitations. LNPs encapsulate aptamers through charge interactions, and surface modification with targeted ligands (such as aptamers themselves) enables dual targeting. While nanocarriers (liposomes and mesoporous silica) can increase tumor enrichment efficiency by 5-fold, interstitial hydraulic pressures exceeding 30 mmHg still restrict deep tumor penetration (120,121). The renal clearance characteristics of nucleic acid aptamers (with a molecular weight <50 kDa) result in insufficient accumulation in RES organs such as the liver and spleen, and their penetration into solid tumors is limited by interstitial hydraulic pressure. The design of dual-targeted PROTACs (combination of AS1411/V7t1) has increased the tumor penetration efficiency to 65% through the dual mechanism of 'vascular leakage-active targeting', offering new insights for solid tumor treatment. Optimizing biodistribution necessitates tailoring molecular structures and delivery strategies to specific treatment scenarios such as a local injection or systemic administration.

Key breakthrough directions for aptamer technology include: i) High-throughput screening and intelligent design: Integrating nanopore sequencing with machine learning has the potential to reduce the SELEX cycle to 7 days and increase the screening success rate by 40%; ii) multimodal collaborative platforms: Developing aptamer-antibody hybrid molecules to combine the Fc-mediated functions of antibodies with the deep penetration capabilities of aptamers (for example, the HER2 aptamer-trastuzumab conjugate has entered preclinical evaluation); and iii) individualized treatment strategies: Customizing aptamers on the basis of patient-specific tumor mutation profiles (or example, a p53 aptamer for TP53-mutated breast cancer is currently undergoing a phase I clinical trial).

Despite these numerous challenges, the application of aptamers in tumor-targeted therapy is becoming increasingly compelling. With advancements in modern biotechnology and aptamer screening methods, designing aptamers tailored to individual tumors and patients is now feasible, paving the way for more precise and personalized treatment. Importantly, this emerging approach is still in its early stages, and further in-depth investigations of numerous parameters are needed. Recent preclinical successes have demonstrated the promise of aptamers, and future clinical research holds the potential to transform breast cancer diagnosis and treatment from a population-based model to an individualized model, offering a revolutionary solution for overcoming tumor heterogeneity and drug resistance.

## Figures and Tables

**Table I:** Aptamer properties and modifications.

Inherent properties	Engineered enhancements	Clinical value anchors
Small size (10-30 kDa)	Phosphorothioate backbone modification	Deep tumor penetration (5-fold higher than antibodies)
Reversible binding	pH-responsive DNA switch	Microenvironment-controlled drug release
Low immunogenicity	20-40 kDa PEGylation	Half-life extended to 6 h
Broad target spectrum	Bispecific design (HER2/EpCAM)	Overcoming tumor heterogeneity

EpCAM, epithelial cell adhesion molecule.

**Table II:** Aptamer-based sensors outperform conventional methods in the quantitative analysis of key biomarkers.

Biomarker	Aptamer-based method	Clinical gold standard	Performance comparison	(Refs.)
Extracellular vesicles	Microfluidic thermophoresis-enhanced aptamer sensor (Cy5-labeled aptamer probes)	Serum proteomic analysis	Diagnostic accuracy: 87.7% (MBC vs. non-MBC vs. healthy donors); 95% confidence interval: 76.3-94.9%	(44,45)
CD44 antigen on circulating tumor cells	C24S aptamer-modified magnetic nanoparticles	Cell Search^®^ System(EpCAM-dependent)	Capture efficiency: >85%	(46)
Estrogen receptor	S1-4 aptamer probe	IHC	Binding affinity Kd=73.6 nM (IHC semiquantitative scoring error >20%)	(47)
Progesterone receptor	RNA aptamer targeting PR DNA binding domain	IHC		(48)
Calreticulin	Laser-induced fluorescent aptamer probe	ELISA	Detection limit of CRT in serum: 0.5 nmol/l (ELISA: 5 nmol/l)	(55)
EGFR	CeO_2_-graphene oxide/aptamer electrochemical sensor	IHC/quantitative PCR	Linear range: 10 fg/ml-100 ng/ml (ELISA: 1 pg/ml-10 ng/ml)	(56)

IHC, immunohistochemistry; MBC metastatic breast cancer; EpCAM, EpCAM, epithelial cell adhesion molecule; PR, progesterone receptor.

**Table III:** The application of aptamers in the diagnosis of breast cancer according to diagnostic method.

A, Liquid biopsy					
Aptamer	Nucleic acid type	Screening approach	Target	Function	(Refs.)
Cy5-conjugated aptamers	DNA	Thermophoresis enrichment of aptamer-bound EVs	EV, breast cancer-related protein markers	EV characteristics have an accuracy of up to 91.1% in distinguishing MBC, non-MBC and healthy donor	(44)
C24S-magnetic nanoparticles	DNA	Cell-SELEX	CD44^+^ cells	The aptamer C24S has high affinity and strong specificity for CD44	(46)
S1-4	DNA	Cell-SELEX	Specifically recognize ER^+^ MCF-7 cells.	S1-4 can target the tumor cell membranes of MCF-7 tumor-bearing mice *in vivo*	(47)
PRapt-3	RNA	CRUSOAK-SELEX	PR	Specific binding to the PR DNA binding domain with high affinity and no cross-reactivity to EGFR, IgE or human serum	(48)
AptaB1, AptaB2, AptaB3, AptaB4 and AptaB5	DNA	Cell-SELEX	MDA-MB-231 MDA-MB-468	AptaB4 and AptaB5 show outstanding performance in recognizing early-stage tumors and metastatic samples	(49)
Apt 23	DNA	CE-SELEX	CRT	FAM-labeled Apt 23 can recognize CRT overexpressed on the surface of breast cancer 4T1 cells	(55)
GCE/CeO_2_-GO/EGFR-Apt/BSA	DNA	Functionalize aptamers through covalent coupling reactions	EGFR	Aptamer can be used forreal-time non-invasive monitoring of EGFR in clinical samples	(56)

B, Imaging examination					

AS1411	DNA	Specifically bind to NCL with high affinity	NCL	Successfully constructed AS1411-nanobubbles. *In vivo*, they can target NCL and prolong the duration of contrast imaging	(62)
MWCNTs-BA-APT	DNA	Covalently conjugate aptamers with MWCNTs-BA via the EDC/NHS	MDA-MB-213	MWCNTs-BA-APT enable effective targeting of MDA-MB-213 cells, featuring good MRI contrast ability	(63)

SELEX, systematic evolution of ligands by exponential enrichment; MBC, metastatic breast cancer; GO, graphene oxide; EV, extracellular vesicles; NCL, nucleolin; CRT, calreticulin; MWCNTs-BA-APT; aptamer-modified multiwalled carbon nanotube; PR, progesterone receptor; EDC, 1-ethyl-3-(3-dimethylaminopropyl)carbodiimide; NHS, N-hydroxysuccinimide; MRI, magnetic resonance imaging; EGFR, epidermal growth factor receptor; GCE, glassy carbon electrode.

**Table IV:** Application of aptamers in the treatment of breast cancer.

A, Drug carriers						
Aptamer	Nucleic acid type	Screening approach	Target	Drug	Function	(Refs.)
AS1411	DNA	-	NCL	Triptolide	The AS1411-triptolde exhibits strong specificity for the MDA-MB-231 cell, and can inhibit tumor growth and induce apoptosis.	(67)
HA1	DNA	Cell-SELEX	HER2	Doxorubicin	Grapefruit-derived nanocarrier-Dox-HA1 can target tumor tissues to inhibit growth in HER2-positive breast cancer cells.	(68)
Anti-EGFR aptamer	DNA	-	EGFR	Noscapine	The aptamer-functionalized nanocomposites can releasedrugs slowly, and exhibit a strong inhibitory effect on MCF-7 cancer cells.	(69)
TA1	DNA	-	TA1 cells	Docetaxel	The drug-loaded micelles constructed have a strong targeting ability and high uptake efficiency for TA1 cells.	(70)

B, Gene therapy						

sTN145	RNA	Cell-SELEX	TNBC cells	siRNA	The sTN145 aptamer can promote the release of siRNA from endosomes, effectively silence the PD-L1 gene and inhibit the expression of related proteins.	(72)
S2.2	DNA	-	MUC1	pLVSIN-iC9	GOQD-PEI/S2.2/pLVSIN-iC9 can efficiently transfect cells, increase the expression of caspase-9, induce cell death and inhibit cell growth.	(73)

C, Immunotherapy						

MUC-1	DNA	-	MUC1	Doxorubicin	In drug-resistant MCF-7/ADR cells, MUC-1 can downregulate the expression of P-gp and efficiently kill cancer cells.	(74)
Gint4.T	RNA	-	TNBC cells/PDGFRβ	Anti-PD-L1 mAB	The combination of the Gint4.T aptamer and anti-PD-L1 mAB can enhance the abilities of inhibiting the proliferation of human and murine TNBC cells.	(79)
PDGC21-T	DNA	Cell-SELEX	TNBC cell	Binds to NK cells	ApEn-NK can specifically bind to both suspended and adherent TNBC cells, efficiently inducing apoptosis and death of TNBC cells.	(80)
AS1411	DNA	-	NCL	Anchored to the surface of M1 macrophages	Construct cholesterol-modified AS1411-anchored CAM1. It has a strong targeting ability toward cancer cells and a notable pro-apoptotic effects.	(81)

SELEX, systematic evolution of ligands by exponential enrichment; NCL, nucleolin; siRNA, small interfering RNA; MUC-1, mucin-1; TNBC, triple-negative breast cancer; PD-L1, programmed death-ligand 1; mAB monoclonal antibodies; NK, natural killer; HER2, human epidermal growth factor receptor 2; EGFR, epidermal growth factor receptor; pLVSIN-iC9, pLVSIN-inducible caspase 9.

## Data Availability

Not applicable.
